# Telemonitoring of children with risk indicators for Autism Spectrum Disorder: preliminary findings

**DOI:** 10.1590/2317-1782/20232021308en

**Published:** 2023-08-14

**Authors:** Alessandra Pinheiro da Silva, Ana Manhani Cáceres-Assenço

**Affiliations:** 1 Programa Associado de Pós-graduação em Fonoaudiologia, Universidade Federal do Rio Grande do Norte - UFRN - Natal (RN), Brasil.

**Keywords:** Child Development, Language Development, Autistic Spectrum Disorder, Telemonitoring, Family

## Abstract

**Purpose:**

monitor the development of pragmatic skills in children with clinical risk indicators for autism spectrum disorder (ASD) before and after the application of an integrated parental guidance protocol.

**Methods:**

Seven families who had children with clinical risk indicators for autism spectrum disorder and were in the diagnostic process participated in this study. The study was divided into three moments: (1) structured interview with parents and assessment of children's pragmatic skills, (2) virtual sessions with guidance to parents related to the characteristics of the condition and skills that can be developed to favor their development, and (3) reassessment of children's pragmatic skills. Statistical analysis considered occupation of communicative space, use of functions and communicative means at ground zero and post-monitoring.

**Results:**

There was no significant difference between the two evaluation moments, but a greater number was observed in the use of communicative acts and more interactive communicative functions as an outcome.

**Conclusion:**

The monitoring of children’s pragmatic skills suggests that they present discrete evolution, especially the more interactive ones, after the application of the integrated parental guidance protocol.

## INTRODUCTION

Human communication encompasses multidimensional aspects for its development and constitutes a strong social instrument. Parents play an important role in this process, since the involvement and affection between caregiver and child can provide a rich environment capable of stimulating the development of communication skills in the first years of life^(1,2)^.

Individuals with autism spectrum disorder (ASD) have persistent impairments in functional communication, associated with a restricted repertoire of interests and activities^(3,4)^. In addition, these individuals may present sensory alterations, impairments in verbal language and difficulties in accepting changes, compromising their participation in activities of daily living^(3)^.

One of the major concerns of families of children with ASD is the development of communication^(3)^. Complaints in this domain are often associated with pragmatics, that is, with the functional use of language. The pragmatic aspects of language develop in early stages and involve verbal and nonverbal aspects. Nonverbal aspects precede speech, and are typically characterized by gestures, looks, vocalizations, babbling, and crying. Pragmatic impairments are common in children with ASD, but are not exclusive to this population^(5)^.

In addition to the various challenges that families face to complete the diagnosis, since 2020 with the pandemic caused by COVID-19 and the need for social distancing, new challenges have emerged in accessing health services^(6)^. At this juncture, alternative methods to face-to-face therapy have been used to optimize the therapeutic process. Among them, the use of telehealth, which incorporates information and communication technologies in distance activities related to different levels of health care, has become increasingly frequent^(7)^.

In this scenario, telemonitoring stands out for allowing remote monitoring of individuals who need not only consultation, but need continuous intervention in a specialized center. In speech therapy practice, in addition to being effective, it can be an important therapeutic alternative in children, due to its low cost. It makes it possible to align the work of the professional with parents and caregivers, the exchange of experiences and makes the family more active in the process.

Considering that parents can collaborate in the identification and monitoring of clinical risk indicators in children with suspected ASD, the use of telemonitoring is an alternative to optimize the diagnostic process and intervention involving social situations in the context of daily life. Its use is promising, even outside the context of social distancing, since remote orientations can reach families in places of difficult access and, thus, favor family empowerment and provide a better prognosis for children^(8)^.

Given the above, this study aimed to monitor the development of pragmatic skills in children with clinical risk indicators for ASD before and after the application of an interdisciplinary parental guidance protocol.

## METHODS

The study procedures were approved by the Research Ethics Committee, under protocol 4,204,936, and all responsible adults signed the Informed Consent Form.

Seven families who were waiting list at the Anita Garibaldi Center for Education and Research in Health (CEPS) of the Santos Dumont Institute (ISD) participated in the study. This is a reference service in the state of Rio Grande do Norte and is authorized by the Ministry of Health to act in the areas of hearing, physical and intellectual disability of children.

The subjects were selected from families cared for in the service with complaints related to speech delay. All underwent an initial integrated evaluation with psychologists, speech-language therapists, physiotherapists and neuropsychologists during the month of September 2020. The inclusion criteria considered were: presenting clinical indicators of risk for ASD; being aged from 24 months; having parents who speak Brazilian Portuguese as their native language and with availability to participate in the proposed weekly orientations. As exclusion criteria were considered the presence of neurological alterations, malformations and/or associated genetic syndromes, uncorrected auditory/visual impairment.

The mean age of the mothers was 32 years, with a minimum of 25 years and a maximum of 42 years. Schooling ranged from elementary school (12.5%) to higher education (12.5%), with the majority having completed high school (75%). Economy class ranged from B1 (25%) to D-E (12.5%). Most of the children were male (87.5%) and at the beginning of the study the mean age of the children was 38 ±4.9 months.

Data collection was performed in three distinct stages. In the first, individual evaluation was performed for clinical characterization of the children, in a single face-to-face meeting lasting about ninety minutes. At this stage, a brief anamnesis with the parents was applied, including a socioeconomic scale^(9)^. Next, the child underwent assessment of pragmatic skills^(10)^. For this, 10 minutes of spontaneous playful interaction between the child and a familiar adult were recorded on video. For analysis, the final five minutes of each recording were considered, recording the shifts of the child and the adult and detailing the use of functions, means and communicative acts.

In the second stage, an integrated parental guidance protocol was applied, with ten sessions held weekly, remotely, for three months. The sessions were conducted in partnership by a speech-language therapist and a neuropsychologist. The digital platforms *Google Meet* and *Whatsapp* were used because they were the most accessible to parents.

This protocol was developed in order to address the main doubts and difficulties of the families that are cared for in the service. The topics covered included the establishment of bond between families, basic concepts about ASD, communicative intention, different means of social communication, the importance of shared attention, of play and social engagement, as well as cognitive functions, storytelling and symbolic play. As it is a reference service in the state, the proposal was to establish an initial reception protocol that could be applied in small groups, viewing to reduce the waiting time. However, it is worth clarifying that families are encouraged to share their demands, with the theme of each session being a starting point for discussions.

Each session had a topic for discussion and contained suggestions for games to optimize the stimulation process in the context of daily life. In addition, the content of the previous sessions was resumed weekly to resolve doubts and identify difficulties of the parents. It is worth noting that the suggestions for activities included resources easily accessible to parents, including recyclable materials. At each session, parents received a digital file with an illustrated summary of the guidelines covered. From these weekly guidelines, at the end of the three months of guidelines, a communicative stimulation booklet was produced for each participant. The sending of these materials occurred by electronic means that each family indicated as preferential.

After the conclusion of the sessions, an individual reassessment of the participants was conducted, but remotely. Parents were instructed to record a video of a playful interaction in a family context. This video should be about 10 minutes long and sent to the researcher. The families were able to clarify their doubts before the recording and did not report difficulties, however the videos sent had an average duration of 5 minutes.

For pragmatic analysis, the variables percentage of occupation of the communicative space, number of communicative acts per minute, number of communicative functions and communicative functions classified as interactive, in addition to the percentage of occupation of verbal, vocal and gestural means were considered. All interactions were independently analyzed by two previously trained speech -language therapists to ensure reliability of the results.

After completing all the steps, the data obtained were submitted to statistical analysis by SPSS software version 21. Descriptive statistical analysis was performed using the median and interquartile range, while inferential analysis was undertaken by the nonparametric Wilcoxon rank test. The significance level adopted was 5%.

## RESULTS

The comparison between the performance before and after the orientation protocol, performed by the nonparametric Wilcoxon signed-rank test, did not reveal statistical differences in the variables related to pragmatic skills. However, the descriptive analysis showed that the median and interquartile range of the second moment were higher for all variables, with the exception of the gestural mean (median and interquartile range) and the occupation of the communicative space (higher median, but lower interquartile range values). This finding suggests that after the orientations there was an improvement in pragmatic performance, due to the use of more communicative acts and functions and higher frequency of vocal and verbal means. In addition, the number of communicative acts per minute had significance close to 5.0%, suggesting a tendency to increase this use after the orientation program (Table 1).

**Table 1:** Comparative analysis of pragmatic performance pre and post parental guidance protocol

Variable	Moment	Median	Interquartile range		P-value
Space occupancy (%)	Pre	41.5	32.3	49.3	0.734
	Post	43.0	31.0	46.0	
Communicative acts per minute	Pre	3.7	2.1	4.6	0.063
	Post	5.0	3.5	5.7	
Communicative functions	Pre	5.0	2.5	5.8	0.176
	Post	6.0	4.0	8.0	
Interactive functions	Pre	1.0	1.0	3.5	0.104
	Post	3.0	1.0	5.0	
Verbal mean (%)	Pre	6.5	0.0	21.3	0.416
	Post	10.0	0.0	29.0	
Vocal mean (%)	Pre	15.0	1.5	30.5	0.176
	Post	30.0	6.0	41.0	
Gestural mean (%)	Pre	86.0	78.5	100.0	0.237
	Post	78.0	64.0	94.0	

statistical difference p<0.05-nonparametric Wilcoxon signed-rank test

## DISCUSSION

The present study investigated the effects of a parental guidance protocol developed for children with clinical indicators of risk for ASD, in addition to investigating in detail pragmatic skills, exploring the main characteristics that are present in this domain.

There was no statistical difference between pragmatic performance before and after the program. However, the descriptive analysis suggests an improvement in the pragmatic profile of these children, especially with regard to the increase in the number of communicative acts, which may be a reflection of the parents realizing that they should follow the child's interest more or waiting longer for a response; in addition to the increase in the use of communicative functions, and reduction in the use of gestural means associated with the discreet increase in the use of vocal and verbal means.

In this perspective, with regard to the general effects of interventions in pragmatics, it is noteworthy that the approaches that integrated a caregiver to the program, through continuing education, demonstrated a very significant effect^(8,11)^. Even when monitoring occurs remotely, there is evidence that the training offered to parents can affect improving social behavior, communication skills and, in addition, a significant effect on the empowerment and knowledge of interventions in ASD^(12)^.

A more recent literature review indicates that most studies using telehealth with this population focus on parents as agents of intervention^(13)^. Videoconferencing through applications with free use is the most common means of contact with participants and a reduction in absences is frequently reported by the authors. It is also interesting to point out that the COVID-19 pandemic accelerated the use of this type of strategy in the evaluation, diagnosis and intervention processes in ASD cases. In summary, the studies report that the use of this technology is viable, provided that the necessary modifications are implemented; in addition to having results equivalent to those of services offered in person^(13)^.

In addition, early interventions mediated by parents allow for continued opportunities for child learning in a variety of situations. With this, the development of children with ASD is enhanced, which favors the generalization of their learning in different environments. It is also noteworthy that when carried out in groups, this type of intervention allows mutual support and the potential reduction of parental stress^(14)^.

In this study, the increase in the proportion of the use of verbal means and the decrease in the use of gestural means coincides with the outcome found in a previous study, and therefore it is possible to observe that the subjects showed an increase in the proportion of communication interactivity after monitoring and guidance to parents^(15)^.

The data regarding the occupation of the communicative space disagree with the study in which the patients occupied much less than 50% of the communicative space^(15)^. This difference, most likely, is related to the fact that in the mentioned study, the records were made with a therapist, while in our case the children interacted with their parents. The literature indicates that individuals with ASD tend to use their communicative skills more in situations of interaction with their family peers^(14)^.

This study has limitations that should be considered, the first of which is the sample size, since a larger number of subjects would strengthen the results. This fact is both due to the characteristics of the subjects enrolled in the waiting list of the service, the sample loss (12.5%, one family) and the moment of pandemic experienced. However, we are clear that in order for the protocol to be replicated and applied on a large scale, a careful analysis of the data is necessary.

Two other important factors that can be scored are^(1)^ the short time interval of orientations, since a follow-up after a few months, which would include the parents' opinion on the communicative development and functionality of the individuals, would be beneficial; and^(2)^ the absence of a control group that would allow the interpretation of the findings as a cause in the outcome and greater control of the variables.

Despite such weaknesses, we can point out contributions of the study, especially for clinical practice, such as the possibility of application in regions with absence of referral services for children with ASD. In addition to making it possible to care for a greater number of families at a low cost, including as an alternative while waiting for a vacancy in the services.

## CONCLUSION

This study investigated the development of pragmatic skills of seven children with clinical indicators of risk for ASD before and after the application of an interdisciplinary protocol of parental guidance via telehealth. Although there was no statistical difference in pragmatic performance between the moments, a slight qualitative improvement was observed in the communicative profile of the studied sample, with an increase in the number of communicative acts and the different communicative functions produced.
