# Supplementary material for: Telemonitoring of children with risk indicators for Autism Spectrum Disorder: preliminary findings
Source: Codas. 2023 Aug 14;35(5):e20210308. doi: 10.1590/2317-1782/20232021308en (PMC10449090; doi:10.1590/2317-1782/20232021308en)
Supplement: Translation

# Translation: Telemonitoramento de crianças com indicadores de risco para Transtorno do Espectro do Autismo: resultados preliminares

**DOI:** 10.1590/2317-1782/20232021308pt

**Authors:** Alessandra Pinheiro da Silva, Ana Manhani Cáceres-Assenço

**Affiliations:** 1 Programa Associado de Pós-graduação em Fonoaudiologia, Universidade Federal do Rio Grande do Norte - UFRN - Natal (RN), Brasil.

**Keywords:** Desenvolvimento Infantil, Desenvolvimento da Linguagem, Transtorno do Espectro Autista, Telemonitoramento, Família

## Abstract

**Objetivo:**

monitorar o desenvolvimento de habilidades pragmáticas em crianças com indicadores clínicos de risco para Transtorno do Espectro do Autismo antes e após a aplicação de um protocolo interdisciplinar de orientação parental.

**Método:**

Participaram deste estudo sete famílias que possuíam filhos com indicadores clínicos de risco para o Transtorno do Espectro do Autismo e estavam em processo diagnóstico. O estudo foi dividido em três momentos: (1) entrevista estruturada com os pais e avaliação das habilidades pragmáticas das crianças, (2) sessões virtuais de orientação aos pais relacionadas às características do Transtorno do Espectro do Autismo e habilidades que podem ser desenvolvidas para favorecer seu desenvolvimento, e (3) reavaliação das habilidades pragmáticas. A análise estatística considerou ocupação do espaço comunicativo, uso de funções e meio comunicativo no marco zero e no pós-monitoramento.

**Resultados:**

Não houve diferença significativa entre os dois momentos de avaliação, mas foi observado maior número no uso de atos comunicativos e funções comunicativas mais interativas como desfecho.

**Conclusão:**

Desta forma o monitoramento das habilidades pragmáticas sugere que estas apresentem discreta evolução, principalmente as mais interativas, após a aplicação do protocolo integrado de orientação.

## INTRODUÇÃO

A comunicação humana abrange aspectos multidimensionais para o seu desenvolvimento e se constitui como um forte instrumento social. Os pais desempenham um papel importante nesse processo, visto que o envolvimento e a afetividade entre cuidador e criança podem propiciar um ambiente rico e capaz de estimular o desenvolvimento das habilidades comunicativas nos primeiros anos de vida^(1,2)^.

Indivíduos com Transtorno do Espectro do Autismo (TEA) apresentam comprometimentos persistentes na comunicação funcional, associado a um repertório restrito de interesses e atividades^(3,4)^. Além disso, estes indivíduos podem apresentar alterações sensoriais, prejuízos na linguagem verbal e dificuldades em aceitar mudanças, comprometendo sua participação em atividades de vida diária^(3)^.

Uma das grandes preocupações das famílias de crianças com TEA é o desenvolvimento da comunicação^(3)^. As queixas neste domínio costumam estar associadas à pragmática, ou seja, ao uso funcional da linguagem. Os aspectos pragmáticos da linguagem se desenvolvem em fases iniciais e envolvem aspectos verbais e não verbais. Os aspectos não verbais precedem a fala, sendo tipicamente caracterizados por gestos, olhares, vocalizações, balbucios e choro. Prejuízos em aspectos pragmáticos são comuns em crianças com TEA, no entanto não são exclusivos dessa população^(5)^.

Em adição aos vários desafios que as famílias enfrentam para concluir o diagnóstico, desde 2020 com a pandemia causada pelo novo coronavírus e a necessidade de distanciamento social, novos desafios surgiram no acesso aos serviços de saúde^(6)^. Nessa conjuntura, métodos alternativos à terapia presencial têm sido utilizados para otimizar o processo terapêutico. Dentre eles, o uso da telessaúde, que incorpora tecnologias da informação e comunicação em atividades à distância relacionadas aos diversos níveis de atenção à saúde, se tornou cada vez mais frequente^(7)^.

Neste cenário, o telemonitoramento destaca-se por permitir o acompanhamento remoto de indivíduos que necessitam não apenas de uma consulta, mas precisam de uma intervenção contínua em um centro especializado. Na prática fonoaudiológica, além de eficaz, pode ser uma importante alternativa terapêutica no público infantil, inclusive por seu baixo custo. Ele possibilita alinhar o trabalho do profissional com os pais e cuidadores, a troca de experiências e torna a família mais ativa no processo.

Considerando que os pais podem colaborar na identificação e monitoramento dos indicadores de risco clínicos em crianças com suspeita de TEA, o uso do telemonitoramento se mostra como uma alternativa para otimizar o processo diagnóstico e a intervenção envolvendo situações sociais no contexto de vida diária. Seu uso é promissor, mesmo em fora do contexto de distanciamento social, visto que as orientações remotas podem alcançar famílias em locais de difícil acesso e, assim, favorecer o empoderamento familiar e proporcionar um melhor prognóstico às crianças^(8)^.

Diante do exposto, este estudo teve como objetivo monitorar o desenvolvimento de habilidades pragmáticas em crianças com indicadores de risco clínicos para o TEA antes e após a aplicação de um protocolo interdisciplinar de orientação parental.

## MÉTODO

Os procedimentos do estudo foram aprovados pelo Comitê de Ética em Pesquisa, sob protocolo 4.204.936, e todos os adultos responsáveis assinaram o Termo de Consentimento Livre e Esclarecido.

Participaram do estudo sete famílias que estavam em fila de espera no Centro de Educação e Pesquisa em Saúde (CEPS) Anita Garibaldi do Instituto Santos Dumont (ISD). Este é um serviço de referência no Estado do Rio Grande do Norte e está habilitado pelo Ministério da Saúde para atuar nas áreas de deficiência auditiva, física e intelectual de crianças.

Os sujeitos foram selecionados a partir das famílias acolhidas no serviço com queixa relacionada a atraso de fala. Todas passaram por avaliação inicial integrada com psicólogos, fonoaudiólogos, fisioterapeutas e neuropsicólogos durante o mês de setembro de 2020. Os critérios de inclusão considerados foram: apresentar indicadores clínicos de risco para TEA; ter idade a partir de 24 meses; ter pais falantes do Português Brasileiro como língua nativa e com disponibilidade para participar das orientações semanais propostas. Como critérios de exclusão foram considerados a presença de alterações neurológicas, malformações e/ou síndromes genéticas associadas, comprometimento auditivo/visual não corrigido.

A idade média das mães foi de 32 anos, sendo a mínima de 25 anos e a máxima de 42 anos. A escolaridade variou entre ensino fundamental (12,5%) e ensino superior (12,5%), com a maioria tendo ensino médio completo (75%). A classe econômica variou entre B1 (25%) e D-E (12,5%). A maioria dos filhos era do gênero masculino (87,5%) e no início do estudo a média de idade das crianças era 38 ±4,9 meses.

A coleta de dados foi realizada em três etapas distintas. Na primeira, foi realizada avaliação individual para caracterização clínica das crianças, em um encontro presencial único com duração de cerca de noventa minutos. Nessa etapa foi aplicada uma breve anamnese com os pais, incluindo a escala socioeconômica da ABEP^(9)^. A seguir, a criança foi submetida à avaliação das habilidades pragmáticas^(10)^. Para isso, foram registrados em vídeo 10 minutos de interação lúdica espontânea entre a criança e um adulto familiar. Para análise, foram considerados os cinco minutos finais de cada gravação, sendo registrados os turnos da criança e do adulto e detalhado o uso de funções, meios e os atos comunicativos.

Na segunda etapa foi aplicado um protocolo integrado de orientação parental, com dez sessões realizadas semanalmente, de forma remota, durante três meses. As sessões eram conduzidas em parceria por uma fonoaudióloga e uma neuropsicóloga. As plataformas digitais *Google Meet* e *Whatsapp* foram utilizadas por serem as mais acessíveis aos pais.

Esse protocolo foi elaborado com o objetivo de abordar as principais dúvidas e dificuldades das famílias que são atendidas no serviço. Os temas abordados incluíram o estabelecimento de vínculo entre as famílias, conceitos básicos sobre o TEA, intenção comunicativa, diferentes meios de comunicação social, a importância da atenção compartilhada, do brincar e do engajamento social, bem como funções cognitivas, contação de histórias e o brincar simbólico. Por se tratar de um serviço de referência no Estado, a proposta foi estabelecer um protocolo de acolhimento inicial que pudesse ser aplicado em pequenos grupos, com vistas a reduzir o tempo em fila de espera. Entretanto, vale esclarecer que as famílias são estimuladas a compartilhar suas demandas, sendo o tema de cada sessão um ponto de partida para as discussões.

Cada sessão possuía um tema para discussão e continha sugestões de brincadeiras para otimizar o processo de estimulação no contexto de vida diária. Além disso, semanalmente era retomado o conteúdo das sessões anteriores para sanar dúvidas e identificar dificuldades dos pais. Vale destacar que as sugestões de atividades incluíam recursos de fácil acesso aos pais, envolvendo inclusive materiais recicláveis. A cada sessão os pais recebiam um arquivo digital com uma síntese ilustrada das orientações abordadas. A partir destas orientações semanais, ao final dos três meses de orientações, foi produzida uma cartilha de estimulação comunicativa para cada participante. O envio destes materiais ocorreu pelo meio eletrônico que cada família indicou como preferencial.

Após a conclusão das sessões, foi conduzida reavaliação individual dos participantes, porém de forma remota. Os pais foram orientados a gravar um vídeo de uma interação lúdica em contexto familiar. Este vídeo deveria ter cerca de 10 minutos e ser enviado à pesquisadora. As famílias puderam esclarecer suas dúvidas antes da gravação e não relataram dificuldades, entretanto os vídeos enviados tiveram duração média de 5 minutos.

Para análise da Pragmática foram consideradas as variáveis percentagem de ocupação do espaço comunicativo, número de atos comunicativos por minuto, número de funções comunicativas e de funções comunicativas classificadas como interativas, além da percentagem de ocupação dos meios verbal, vocal e gestual. Todas as interações foram analisadas, de forma independente, por duas fonoaudiólogas treinadas previamente, para garantir confiabilidade aos resultados.

Após concluídas todas as etapas, os dados obtidos foram submetidos à análise estatística pelo software SPSS versão 21. A análise estatística descritiva foi realizada por meio da mediana e do intervalo interquartil, enquanto a análise inferencial foi empreendida pelo teste não paramétrico de postos de Wilcoxon. O nível de significância adotado foi de 5%.

## RESULTADOS

A comparação entre o desempenho pré e pós o protocolo de orientação, realizada pelo teste não paramétrico de postos de Wilcoxon, não revelou diferenças estatísticas nas variáveis relacionadas às habilidades pragmáticas. Entretanto, a análise descritiva evidenciou que a mediana e o intervalo interquartil do segundo momento foram maiores para todas as variáveis, com exceção do meio gestual (mediana e intervalo interquartil) e da ocupação do espaço comunicativo (maior mediana, porém menores valores de intervalo interquartil). Esse achado sugere que após as orientações houve melhora no desempenho pragmático, devido ao uso de mais atos e funções comunicativas e maior frequência dos meios vocal e verbal. Ademais, o número de atos comunicativos por minuto teve significância próxima a 5,0%, sugerindo uma tendência ao aumento deste uso após o programa de orientação (Tabela 1).

**Tabela 1:** Análise comparativa entre o desempenho pragmático pré e pós protocolo de orientação parental

Variável	Momento	Mediana	Intervalo interquartil		Valor de p
Ocupação espaço (%)	Pré	41,5	32,3	49,3	0,734
	Pós	43,0	31,0	46,0	
Atos comunicativos por minuto	Pré	3,7	2,1	4,6	0,063
	Pós	5,0	3,5	5,7	
Funções comunicativas	Pré	5,0	2,5	5,8	0,176
	Pós	6,0	4,0	8,0	
Funções interativas	Pré	1,0	1,0	3,5	0,104
	Pós	3,0	1,0	5,0	
Meio verbal (%)	Pré	6,5	0,0	21,3	0,416
	Pós	10,0	0,0	29,0	
Meio vocal (%)	Pré	15,0	1,5	30,5	0,176
	Pós	30,0	6,0	41,0	
Meio gestual (%)	Pré	86,0	78,5	100,0	0,237
	Pós	78,0	64,0	94,0	

Diferença estatística p<0,05 - teste não paramétrico de postos de Wilcoxon

## DISCUSSÃO

O presente estudo investigou os efeitos de um protocolo de orientação parental elaborado de forma integrada para crianças com indicadores clínicos de risco para TEA, além de investigar de forma detalhada as habilidades pragmáticas, explorando as principais características que estão presentes nesse domínio.

Não houve diferença estatística entre o desempenho pragmático antes e após o programa. Porém, a análise descritiva sugere melhora no perfil pragmático destas crianças, especialmente no que se refere ao aumento do número de atos comunicativos, que pode ser reflexo dos pais percebendo que devem seguir mais o interesse da criança ou aguardando mais por uma resposta; além do aumento no uso de funções comunicativas, e redução do uso do meio gestual associado ao discreto aumento do uso dos meios vocal e verbal.

Nessa perspectiva, com relação aos efeitos gerais das intervenções em pragmática destaca-se que as abordagens que integraram um cuidador ao programa, por meio de educação continuada, demonstraram um efeito bastante significativo^(8,11)^. Mesmo quando o monitoramento ocorre de forma remota, há evidências de que o treinamento oferecido aos pais pode ter efeito na melhoria do comportamento social, nas habilidades de comunicação e, para além disso, um significativo efeito no empoderamento e conhecimentos das intervenções no TEA^(12)^.

Uma revisão de literatura mais recente indica que a maioria dos estudos que utilizam telessaúde com essa população focam nos pais como agentes da intervenção^(13)^. Videoconferências por aplicativos com uso gratuito são o meio mais comum de contato com os participantes e uma redução nas faltas é reportada com frequência pelos autores. É interessante ainda pontuar que a pandemia de COVID-19 acelerou o uso desse tipo de estratégia nos processos de avaliação, diagnóstico e intervenção em casos de TEA. Em síntese, os estudos reportam que o uso dessa tecnologia é viável, desde que as modificações necessárias sejam implementadas; além de ter resultados equivalentes aos de serviços ofertados de forma presencial^(13)^.

Ademais, intervenções precoces mediadas pelos pais permitem oportunidades contínuas para o aprendizado da criança em uma variedade de situações. Com isso, o desenvolvimento de crianças com TEA é potencializado, o que favorece a generalização de sua aprendizagem em diferentes ambientes. Destaca-se ainda que quando realizada em grupos, esse tipo de intervenção permite o apoio mútuo e a redução potencial do estresse parental^(14)^.

Neste estudo, o aumento na proporção do uso do meio verbal e a diminuição do uso do meio gestual, coincide com o desfecho encontrado em um estudo anterior, sendo, portanto, possível observar que os sujeitos apresentaram aumento na proporção da interatividade da comunicação após monitoramento e orientações aos pais^(15)^.

Os dados referentes à ocupação do espaço comunicativo discordam do estudo em que os pacientes ocupavam bem menos que 50% do espaço comunicativo^(15)^. Essa diferença, provavelmente, está relacionada ao fato de que, no estudo mencionado, os registros foram feitos com um terapeuta, enquanto no nosso caso as crianças interagiam com seus pais. A literatura aponta que indivíduos com TEA tendem a utilizar mais as suas habilidades comunicativas em situações de interação com seus pares familiares^(14)^.

Este estudo apresenta limitações que devem ser consideradas, a primeira delas é o tamanho da amostra, visto que um número maior de sujeitos fortaleceria os resultados. Esse fato é tanto decorrente das características dos sujeitos inscritos na lista de espera do serviço, da perda amostral (12,5%, uma família) e do momento de pandemia vivenciado. Porém, temos clareza de que para que o protocolo possa ser replicado e aplicado em larga escala faz-se necessário uma análise cautelosa dos dados.

Outros dois fatores importantes que podem ser pontuados são^(1)^ o curto intervalo de tempo de orientações, visto que um acompanhamento após alguns meses, e que incluísse a opinião dos pais sobre o desenvolvimento comunicativo e a funcionalidade dos indivíduos, seria benéfico; e^(2)^ a ausência de grupo controle que possibilitaria a interpretação dos achados como causa no desfecho e maior controle das variáveis.

Apesar de tais fragilidades, podemos apontar contribuições do estudo, principalmente para prática clínica, como a possibilidade de aplicação em regiões com ausência de serviços de referência a crianças com TEA. Além de possibilitar atender um maior número de famílias por um baixo custo, inclusive como alternativa durante a espera por vaga nos serviços.

## CONCLUSÃO

Este estudo investigou o desenvolvimento das habilidades pragmáticas de sete crianças com indicadores clínicos de risco para o TEA antes e após a aplicação de um protocolo interdisciplinar de orientação parental via telessaúde. Apesar de não haver diferença estatística no desempenho pragmático entre os momentos, foi observada discreta melhora qualitativa no perfil comunicativo da amostra estudada, com aumento do número de atos comunicativos e das diferentes funções comunicativas produzidas.
